# Competitive metabolism of *L*-arginine: arginase as a therapeutic target in asthma^☆^

**DOI:** 10.1016/S1674-8301(11)60041-9

**Published:** 2011-09

**Authors:** Jennifer M. Bratt, Amir A. Zeki, Jerold A. Last, Nicholas J. Kenyon

**Affiliations:** Department of Internal Medicine, Division of Pulmonary and Critical Care and Sleep Medicine, University of California, Davis, CA 95616, USA

**Keywords:** nitric oxide, *L*-arginine, arginase, nor-NOHA, nitrosation, nitric oxide synthase

## Abstract

Exhaled breath nitric oxide (NO) is an accepted asthma biomarker. Lung concentrations of NO and its amino acid precursor, *L*-arginine, are regulated by the relative expressions of the NO synthase (NOS) and arginase isoforms. Increased expression of arginase I and NOS2 occurs in murine models of allergic asthma and in biopsies of asthmatic airways. Although clinical trials involving the inhibition of NO-producing enzymes have shown mixed results, small molecule arginase inhibitors have shown potential as a therapeutic intervention in animal and cell culture models. Their transition to clinical trials is hampered by concerns regarding their safety and potential toxicity. In this review, we discuss the paradigm of arginase and NOS competition for their substrate *L*-arginine in the asthmatic airway. We address the functional role of *L*-arginine in inflammation and the potential role of arginase inhibitors as therapeutics.

## INTRODUCTION

Asthma is a common disease characterized by a syndrome of persistent airway inflammation and reversible airway obstruction. Intermittent obstruction of the airways results from influx of inflammatory cells, increased mucus secretion, edema, and airway smooth muscle constriction. Chronic inflammation leads to long term remodeling of the lung including mucus cell hyperplasia and metaplasia[1], smooth muscle hyperplasia[1],[2], and increased basement membrane thickness from accumulation of collagens in the submucosal and reticular basement membrane[3]. The airway remodeling and resultant reduction in overall lung function can become irreversible.

Current methods of diagnosing asthma and assessing patient response to therapy are inexact and include measuring lung function with spirometry and assessing noninvasive exhaled breath biomarkers[4] and expectorated sputum samples. One biomarker present in higher concentrations in the exhaled breath of asthmatics, exhaled nitric oxide (NO), has been positively correlated with lung inflammation severity. However, clinical trials with inhibitors targeting the NO producing enzymes have produced mixed results[5],[6], indicating that the role of NO during asthma exacerbation or mediation is much more complex than previously thought.

Derived primarily from the metabolism of *L*-arginine by the NO synthase (NOS) family of enzymes, NO is essential in preserving normal lung function. The NO diffusion gradient ensures sufficient blood oxygenation by dilating vascular smooth muscle at regions of hypoxia, thereby maintaining proper ventilation-perfusion matching[7]–[9]. NO also regulates the ciliary beat frequency[10] of columnar epithelial cells in the airway that clear potentially obstructive agents, including foreign materials and mucus from the upper conducting airways. As an inhibitory non-adrenergic non-cholinergic (iNANC) signaling molecule[11],[12], NO controls smooth muscle tone in the airways by activating the soluble guanylate cyclase in the smooth muscle[13]. NO modulates inflammation by affecting leukocyte adhesion to the endothelium[14],[15] and vascular permeability[16] and also is an integral part of the immune system anti-microbial arsenal, reacting with other reactive species to form potent oxidant molecules[17],[18].

Thus, despite the correlation of increased exhaled NO with inflammatory severity in the lung[4],[19], reducing the overall production of NO by inhibiting NOS enzymes would undoubtedly also affect NO-dependent regulation of normal lung function. The variability in outcomes using NOS inhibitors in animal models of allergic inflammation supports the conclusion that not all sources of NO are equal (See Mathrani, *et al*. 2007 for review of NOS inhibition in allergic asthma models[20]). Focusing entirely on regulating a measurable parameter, exhaled NO, does not take into account the sources of NO production or the delicate balance of NO in the lung as a whole. The more telling question may be whether there is “good NO” and “bad NO”, what their cellular sources are, and what changes occur in the lung during allergic inflammation that affect both “good” and “bad” NO.

## THE FUNCTIONAL ROLE OF NO AND ITS PRESURSOR, *L*-ARGININE

### Nitric oxide: function and form interdependence

The NO molecule is a neutral-charged free radical with a short half life in biological fluids (<1 ms) due to its reactivity with surrounding proteins, free radical species, and reducing molecules of the intra- and extracellular compartments like glutathione. NO is primarily derived from the enzymatic conversion of the amino acid *L*-arginine and molecular oxygen into NO and citrulline by the NOS family of enzymes.

The NOS enzyme family is comprised of three isoforms, NOS1, NOS2 and NOS3, which vary in their regulatory mechanism and tissue expression patterns[21]. NOS1 and NOS3 are constitutive NOS enzymes that require intracellular calcium/calmodulin binding for activation. In addition to a calcium concentration dependence, NOS3 activity is also regulated by multi-site phosphorylation of serine and threonine residues[22]. NOS2, the inducible NOS, is predominantly regulated at the transcriptional level. Due to its high affinity for calmodulin, NOS2 activity is relatively independent of intracellular calcium fluxes but requires binding of transcriptional activators nuclear factor-kappa B(NF-κB), activator protein-1 (AP-1) or signal transducers and activators of transcription 1α STAT1α[23]–[25] for expression. The NOS2 isoform can be rapidly induced by pro-inflammatory cytokines, resulting in heightened levels of NOS2 protein expression and NO production; thus, NOS2 can become the major source of NO under inflammatory conditions.

The three NOS isoforms are differentially expressed in numerous resident and inflammatory cell types in the lung and can vary in both expression and activity under normal and proinflammatory conditions. NOS1 is expressed mainly in airway epithelial cells[26] while NOS3 is expressed in the airway epithelium and vascular endothelium[27]. NOS1 and NOS3 are both expressed under basal conditions and contribute to the baseline concentrations of exhaled NO. NOS2 is expressed at low to undetectable levels under non-inflammatory conditions but can be expressed at high levels in the airway epithelium, airway smooth muscle, inflammatory cells and alveolar type 2 cells under inflammatory conditions. NOS2 is thought to contribute to the increase in exhaled NO observed in asthmatics and animal models of allergic inflammation. Despite tight regulatory controls over the constitutive NOS1 and NOS3 isoforms, NOS2 isoform expression can change depending on surrounding NO concentration and cytokine expression[28]. As a result, NO production by the different enzymatic isoforms can vary significantly depending on the surrounding conditions and have sweeping effects on lung function.

The rate of clearance of NO also depends on numerous factors. Accumulation in protected cellular compartments, including the plasma membrane, lipophilic protein folds and interstitial spaces (the inner mitochondrial space or vesicles) can increase the half-life of the molecule[29]. Reaction of NO with glutathione, forming S-nitrosoglutathione (GSNO), or with albumin or hemoglobin can convert NO into a more stable intermediate, giving NO the capacity to have functional activity far removed from its temporal and positional origin. The oxidization products of NO, nitrate and nitrite, are more stable than NO and can serve as a substrate pool for NO under hypoxic conditions by enzymatic conversion using xanthine oxidoreductase[30] or by non-enzymatic reduction via electron and proton transfer reactions with both free and protein-associated heme[31],[32]. Excessive nitrate and nitrite can be filtered from the plasma and excreted in the urine or exhaled from the lung directly as either NO or as one of its many oxidation products.

### *L*–arginine and inflammation

NO production can be greatly increased under inflammatory conditions but, like other enzymatic reactions, is limited by the amount of active enzyme present, the concentration of the enzymatic cofactor, tetrahydrobiopterin (BH4), and of the substrate, *L*-arginine. *L*-arginine is a semi-essential amino acid that serves as a substrate for numerous enzymatic pathways and a precursor for protein synthesis[33]. In the body, circulating *L*-arginine concentration in the plasma is the sum of the dynamic interconversion of *L*-arginine downstream metabolites, protein synthesis and degradation, dietary intake and excretion. *L*-Arginine is first absorbed in the gut through the epithelium of the small intestine where it is converted into *L*-citrulline and then enters the circulation. Synthesis of *L*-arginine from *L*-citrulline occurs mainly in the kidney by the concerted enzymatic activities of the argininosuccinate synthase (AS) and argininosuccinate lyase (AL), although certain cell types, including alveolar macrophages, retain the capacity to regenerate *L*-arginine by this pathway.

*L*-arginine incorporated into proteins can be posttranslationally modified by methyltransferases, yielding asymmetric dimethylarginine (ADMA), symmetric dimethylarginine (SDMA) and N-monomethylarginine (*L*-NMMA). After protein degradation, these methylated products are released back into the free amino acid pool[34],[35] where they can competitively inhibit NOS activity and compete with *L*-arginine for transmembrane transport by the cationic amino acid transporter (CAT). The dimethylarginine molecules can also be enzymatically converted back into *L*-arginine by dimethylarginine dimethylaminohydrolase (DDAH) or excreted in the urine[36].

**Fig. 1:**
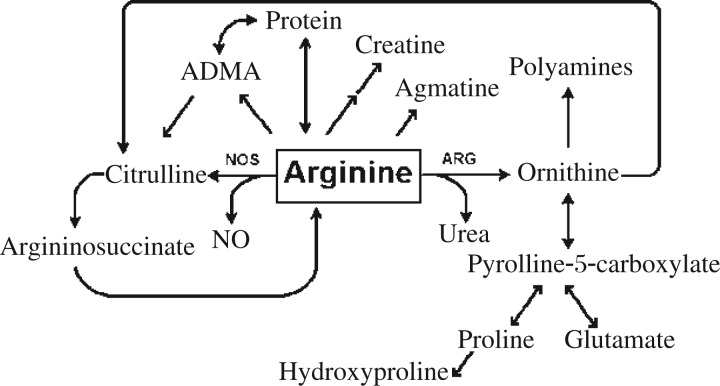
Schema of major *L*-arginine metabolic pathways

*L*-arginine is also metabolized by several different metabolic pathways, resulting in the formation of downstream products such as creatine, agmatine, glutamate, proline, polyamines, ornithine and NO (***Fig. 1***). These metabolic products can affect the development of or perpetuate the asthmatic phenotype. Creatine has been linked to increased airway hyperreactivity as measured by Penh and increased airway eosinophilia, though only at high supplemented dosages[37]. Agmatine is a weak NOS inhibitor[38] and glutamine is a precursor for γ-aminobutyric acid (GABA) and glutathione, which increases mucus production in the airway epithelium or act as an antioxidant, respectively[39],[40]. Proline is a precursor for collagen, a component of the basement membrane that becomes thickened during asthmatic airway remodeling[41],[42]. Polyamines are important regulators of the cell cycle, proliferation, differentiation and apoptosis[41]–[43].

With systemic or chronic inflammation, the rate of *L*-arginine reconversion may become insufficient to maintain normal plasma concentration[44]–[46]. In this case, total *L*-arginine catabolic and anabolic reaction rates have become unbalanced, producing an overall shift toward *L*-arginine catabolism. This *L*-arginine flux imbalance may occur throughout the whole body, reducing overall circulating *L*-arginine, be limited to a tissue microenvironment where the diffusion of *L*-arginine from the circulating plasma to the target tissues becomes the limiting factor, or occur within specific cellular compartments where diffusion and transmembrane transport rates may affect *L*-arginine supply[47]–[49].

Reduced *L*-arginine content has been detected in the airway compartment of mice exposed to ovalbumin (OVA) to mimic allergic airway disease[50],[51]. In moderate asthmatic subjects, circulating plasma *L*-arginine is reduced compared to control subjects (45 µmol/L *L*-arginine compared to control values of 94 µmol/L). The decrease in plasma *L*-arginine in asthmatics coincides with a 3-fold increase in plasma arginase activity[52] and occurs concurrently with changes in the dynamics of *L*-arginine's primary metabolic pathways, the NOS pathway and the arginase pathway.

Expression of arginase I (Arg1) increases in murine models of allergic asthma induced by either OVA, *A. fumigatis* exposure[53]–[56], or house dust mite allergen (HDMA)[57] (up to an 8-fold increase in total Arg1 expression in the airways of mice exposed to OVA). The increase in Arg1 expression in mice directly correlates with increased arginase activity[56]. This change in *L*-arginine metabolism is thought to control NOS-dependent production of NO by the depletion of their common substrate, *L*-arginine.

### *L*-arginine depletion: enzyme uncoupling

The depletion of *L*-arginine in the airways can have consequences in addition to reducing total NO production. Constitutive NOS isoforms perform a two step conversion of *L*-arginine and oxygen to *L*-citrulline and NO. In the absence of sufficient *L*-arginine (under 100 µmol/L) and/or BH_4_, NOS activity can produce superoxide (•O_2_^—^). This is termed “uncoupling” as the oxygenase domain is uncoupled from the reductase domain of the enzyme[58]. Uncoupling results in the constitutively active NOS enzymes producing a combination of NO and superoxide[59]–[61], which can combine to form peroxynitrite.

The uncoupling phenomenon only occurs at low BH4 concentration in the inducible NOS isoform[62]. Examination of the catalytic mechanism of the NOS2 enzyme identified the flavin-binding reductase domain, not the oxygenase domain, of NOS2 as the source of superoxide production[63]. This difference in catalytic mechanism in the NOS2 isoform, as contrasted with the constitutive NOS1 and NOS3 isoforms, may exist because the antimicrobial action of NO depends on its reaction with the free radical species superoxide to form the more potent oxidant peroxynitrite.

### Arginase: a competitor for *L*-arginine

The arginase enzymes are homotrimeric metalloenzymes stabilized in conformation by two Mn^2+^ ions per monomeric structure. There are two isoforms of arginase, Arg1 and arginase 2 (Arg2). Arg1 is often referred to as liver arginase, especially in older texts, as it is found at high levels in the liver as part of the urea cycle of enzymes, but it is also expressed in bone marrow-derived cells[64], such as neutrophils, macrophages[65] and red blood cells in humans, as well as in fibroblasts[66], epithelial and endothelial cells[67] upon activation by proinflammatory cytokines or hypoxia. Arg2 is found ubiquitously at low levels in mitochondria and at high levels in the kidney.

Although Arg1 and NOS2 are expressed in the airway and inflammatory cells of the lung, and in some cases within the same cell, there is controversy regarding the ability of arginase to compete with NOS for *L*-arginine due to kinetic properties of the two enzymes. Kinetic assays using isolated arginase have indicated the maximum rate of hydrolysis of *L*-arginine into *L*-ornithine and urea at a *Km* of 1 mmol/L and a *Vmax* of 4,380 µmol/(min·mg)[68]. Based solely upon the calculated *Km* values, (Arg: 1 mmol/L, and NOS: 10 µmol/L), the arginase enzymes do not appear capable of competing with the NOS enzymes at *L*-arginine concentrations below 100 µmol/L. However, the enzymes' kinetic parameters were determined in a closed system with isolated enzymes and do not take into account enzyme coupling, non-freely diffusible substrate pools, intracellular localization of the enzymes and substrate transporter expression and activity, diffusion gradients, and potential sequestration[69]–[72].

*In vitro* studies in isolated endothelial cells note that increasing the extracellular arginine concentration increases NO production despite average intracellular *L*-arginine concentrations than would be considered saturating for the enzyme based upon its kinetics. Colocalization of cationic amino acid transporters (CAT) 1 and 2B, and *L*-arginine recycling enzymes, AS and AL, with NOS3 make the NOS3 enzyme dependent upon extracellular *L*-arginine concentration, the concentration of other cationic amino acids that can compete for transport via CAT, and/or the rates of *L*-arginine re-synthesis[73],[74].

In the 1990s, research began to focus on the potential for arginase activity to regulate airway reactivity. In a series of experiments utilizing *ex vivo* airway preparations from allergen-exposed guinea pigs, de Boer *et al*. observed the dependence of airway hyperreactivity on NO deficiency[75],[76] and that this deficiency was *L*-arginine concentration dependent[77]. By analyzing NO production in the presence and absence of the arginase inhibitor Nω-hydroxy-nor-arginine (nor-NOHA), researchers showed that the deficiency of NO caused by arginase activity was the causative agent for the development of airway hyperreactivity[78]–[81]. Although this mechanism was previously identified in cultured macrophages[82],[83] and endothelial cells[84], the identification of this phenomenon in the resident airway cells of the lung had a significant impact on the understanding of NO function in the airways.

**Fig. 2:**
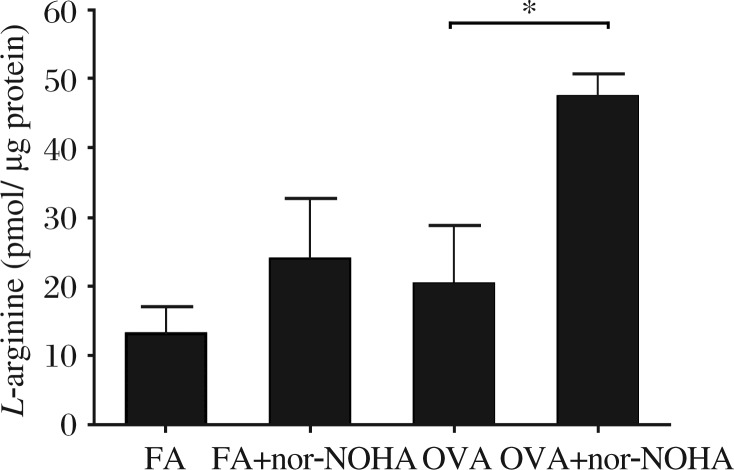
*L*-arginine content in microdissected airways of BALB/c mice sensitized and exposed to ovalbumin (OVA) for two weeks. Mice exposed to ovalbumin and treated with the arginase inhibitor Nω-hydroxy-nor-*L*-arginine (nor-NOHA) had higher levels of *L*-arginine in their dissected airways compared to mice exposed to ovalbumin alone. FA: filtered air. *P < 0.05.

Recently, we have found that systemic treatment of mice with a competitive inhibitor of arginase in a model of allergen-induced airway inflammation significantly increased the amount of NO produced, as measured by the nitrite + nitrate (NOx) concentration in lung lavage supernatant[50]. We also verified that arginase inhibition increases *L*-arginine content in the conducting airways of C57BL/6 mice exposed to OVA (***Fig. 2***). Examination of Arg1 expression in OVA-exposed C57BL/6 and congenic NOS2 knockout mice[1] demonstrated that Arg1 content in the airway compartment may be regulated by NOS2 activity. Expression levels of Arg1 also vary between BALB/c mice and C57BL/6 strains[50],[56], but these strain differences may be linked to variability in NF-κB signaling which is altered in the two strains[85] and in the NOS2 knockout mice. Given the apparent linked regulation of Arg1 and NOS, targeted therapeutic interventions at one of these enzymes will be needed to recognize and account for the effects on the other.

### *L*-arginine and arginase: potential therapeutic targets

In addition to asthma, arginase activity has been shown to correlate with the disease severity in several lung diseases including COPD, cystic fibrosis and sickle cell anemia[86],[87]. To better understand the role of arginase activity in perpetuating and/or potentiating inflammatory lung disease, arginase inhibitors have been used extensively in animal models of allergen-induced inflammatory lung disease using tracheal explants and in cell culture. In these models, the inhibition of arginases increases the production of NO, the product of the competing NOS pathway.

The liver and kidneys contribute a significant portion of the body's overall arginase activity and prolonged treatment with arginase inhibitors may inadvertently target these highly perfused organ systems causing metabolic imbalance. Tissue-specific targeting of arginase inhibitors would limit systemic effects, but can be difficult to achieve due to the hydrophilic nature of the currently available drugs, which are mainly structural analogues of arginine[88].

**Fig. 3:**
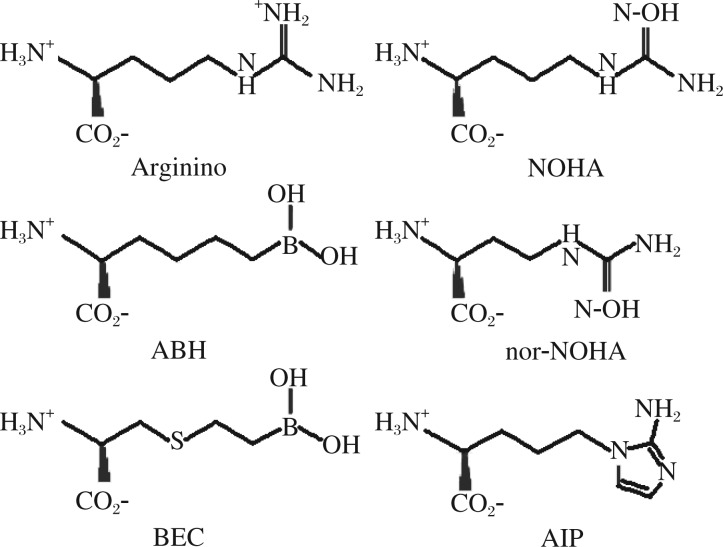
Key arginase inhibitors of therapeutic interest in asthma. Adapted from a research of Morris SM Jr. [88]

There are two basic subsets of arginase inhibitors: reversible and irreversible. The reversible inhibitors can be further divided into boronic acid-based inhibitors and non-boronic acid-based inhibitors. Most reversible inhibitors are *L*-arginine molecular analogs, which mimic the transition-state structure of *L*-arginine during the arginase hydrolysis reaction (***Fig. 3***). The inhibitors are structurally similar to the molecule, Nω-hydroxy-*L*-arginine (NOHA), an intermediate in the synthesis of NO by the NOS family of enzymes. Although NOHA can act as an arginase inhibitor and a substrate for NOS, transition state inhibitors, such as nor-NOHA and N(G)-hydroxy-*L*-arginine (*L*-NOHA) cannot be converted to NO by the NOS enzymes or inhibit NOS enzyme activity. In addition to the NOHA-based molecules, there is a small group of amino acid sulfonamides that can inhibit arginase activity[89]. These compounds are also transition state mimetics with an activated guanidino group that bridges the binuclear manganese cluster, but with a lower binding efficiency than the boronic and NOHA-based inhibitors.

2-(S)-amino-5-(2-aminoimidazol-1-yl)pentanoic acid (A1P) is a recently developed arginase inhibitor that utilizes a 2-aminoimidazole moiety in place of a guanidine side chain (***Fig. 3***). This newly developed arginase inhibitor has a *Ki* of 4 µmol/L and has recently been used in a murine model of allergic inflammation to significantly reduce airway hyperresponsiveness[90]. Much like the non-boronic acid based inhibitors, the boron-based arginase inhibitors are also transition state inhibitors but with a significantly higher potency, ≥5-fold lower IC_50_ and 5-fold lower *Ki*, and cannot serve as a substrate for NOS enzymes[91],[92]. Boron-based inhibitors, 2(S)-amino-6-boronohexonic acid (ABH) or S-(2-boronoethyl)-*L*-cysteine (BEC), have been used extensively in animal models of allergic asthma with considerable success[79],[93]–[95].

The irreversible arginase inhibitor (+)-S-2-amino-6-iodoacetamidohexanoic acid (2-AIHA) is also a potent inhibitor of the enzyme. 2-AIHA is an Nω-derivative of (+)-lysine with significant toxicity issues, including liver and kidney damage in mice[96].

Several other classes of drugs can also affect arginase indirectly by acting as a general anti-inflammatory agent: NSAIDs, corticosteroids, and statins – the latter two of which block Arg1 induction in murine and human tissues[66],[97],[98]

Several investigators have taken the information from previously described *in vitro* and *ex vivo* studies to examine animal models of allergic airway disease in order to better understand the complex relationship between NO, airway hyperreactivity and airway inflammation. Experiments utilizing arginase inhibitors and Arg1 RNAi have indicated that arginase inhibition can decrease airway inflammation and reduce airway hyperreactivity[56],[93],[94],[99]. However, a study by Ckless et al.[100] noted an increase in airway inflammation and hyperresponsiveness, with significant increases in nitrotyrosine and S-nirosylation, indicating an increase in NO-based oxidative products. In this study, mice were treated with the inhibitor using a single intratracheal dose 2 h after the final (3^rd^) allergen exposure. Although oxidative products increased with a single dose, there were reductions in IL-4; thus, the increase in oxidative stress products may have been due to preestablished uncoupling of the NOS enzymes in the lung.

Supplementation of *L*-arginine or manipulation of NOS expression has shown similar effects on airway hyperreactivity and inflammation, further supporting the theory that increasing NOS substrate concentration, either by manipulation of competing metabolic pathways or increasing substrate concentration, directly impacts upon the severity of the allergic response[93],[101]. For example, the deletion of the NOS2 isoform increases the influx of inflammatory cells and airway hyperreactivity, whereas overexpression of NOS3 reduces these parameters[102]–[104]. It is interesting that both Ten Broeke *et al*[103] and Kobayashi *et al*[104] found that a strategy of overexpressing NOS3 in the vascular and airway epithelium of mice to increase NO levels decreased allergic airway inflammation, chemokine expression, and airway hyperrsponsiveness. Providing substrate to the “right” NOS isoform is appealing therapeutically, but clearly a challenge. For example, we found that supplementation of asthmatic patients with relatively high doses of *L*-arginine (5-8 g/day) in order to increase substrate availability to the NOS enzymes gives unpredictable results. Some asthmatic subjects boost exhaled NO levels, while others have a significant increase in the downstream products of arginase, as measured in serum. Furthermore, supplementation in asthmatic patients may cause short term increases in plasma ADMA[105], which may result from increased efflux of intracellular ADMA into the plasma compartment. While the strategy of *L*-arginine supplementation is appealing because it is inexpensive and readily available, it will be important to discover a biomarker or NOS/arginase genotype that predicts some response to therapy.

The use of arginase inhibitors and of *L*-arginine administration as a therapeutic intervention is not limited to models of allergic asthma. These approaches have also been used successfully in models of ischemia–reperfusion injury[106]–[109], endothelial dysfunction[110]–[112], and *Leishmania* infection[113],[114], all of which share the commonality of arginase depletion of *L*-arginine.

## CONCLUSION

The current paradigm on the interaction between arginase and NOS enzymes in allergic airway inflammation and hyperreactivity is that increased NOS and arginase activity results in competition between these two metabolic pathways for the common substrate, *L*-arginine. The competition between the ARG and NOS does not necessarily occur solely through the ARG1 and NOS2 isoforms, as this has been shown to vary depending on the tissue and disease state of interest[106]–[116]. The increase in *L*-arginine metabolism that accompanies allergic inflammation can lead to the depletion of *L*-arginine, which may be limited to the lung tissue or cause depletion of circulating plasma *L*-arginine. Supplementation of *L*-arginine and/or arginase inhibition can reduce the impact of the arginase metabolic pathways on NO production, reducing the severity of airway inflammation and the development of airway hyperreactivity.
